# Computed tomography for the detection of myocardial hypoperfusion in acute myocardial infarction and the associated CT-to-catheter time

**DOI:** 10.1038/s41598-024-75499-7

**Published:** 2024-10-18

**Authors:** Karim Mostafa, Hatim Seoudy, Schekeb Aludin, Domagoj Schunk, Hannes Peckolt, Carmen Wolf, Mohammed Saad, Marcus Both, Olav Jansen, Derk Frank, Patrick Langguth

**Affiliations:** 1 Department of Radiology and Neuroradiology, University Medical Center Schleswig Holstein, Campus Kiel, Arnold-Heller-Street 3, 24105 Kiel, Germany; 2 Department of Internal Medicine III, Cardiology, Angiology and Critical Care, University Hospital Schleswig Holstein, Kiel, Germany; 3 DZHK (German Centre for Cardiovascular Research), Partner Site Hamburg/Kiel/Lübeck, Kiel, Germany; 4 Interdisciplinary Emergency Department, University Medical Center Schleswig-Holstein, Campus Kiel, Kiel, Germany

**Keywords:** Acute myocardial infarction, Myocardial hypoperfusion, CT perfusion defect, Invasive coronary angiography, Myocardial perfusion, Interventional cardiology, Acute coronary syndromes, Myocardial infarction, Diagnostic markers

## Abstract

Emergency computed tomography (CT) often does not allow for comprehensive coronary artery assessment. However, CT may reveal pathological myocardial hypoperfusion suggestive of acute myocardial infarction (AMI), especially in patients presenting with a different diagnostic hypothesis. CT hypoperfusion is known to be associated with myocardial infarction, however the diagnostic value of CT hypoperfusion for the detection of AMI is still not well evaluated. This was a single-centre retrospective study including patients who underwent invasive coronary angiography (ICA) due to suspected AMI based on incidental perfusion defects upon emergency CT imaging between 2018 and 2023. A total of 22 patients (mean age 66.3 ± 10.8 years, 11 female) were included in this analysis. The diagnosis of AMI was established in all cases leading to ICA. Culprit coronary artery lesions with an indication of percutaneous coronary intervention were detected in all patients who underwent ICA. Spearmann correlation for hypoperfused segments on CT imaging and the corresponding vascular territory upon ICA was significantly substantial (ρ = 0.73, p = < 0.001). The higher the number of affected myocardial segments, the faster ICA was initiated. Mean time between the suspicion of AMI on CT imaging and ICA was 196 (29–4044) minutes. Myocardial hypoperfusion on emergency CT imaging should be considered as AMI until proven otherwise, independent of the clinical scenario leading to performance of CT imaging and whether imaging was performed for the exclusion of non-cardiac pathologies. Early initiation of further diagnostic workup may potentially avoid delays to invasive treatment and reduce the CT-to-catheter-time. Our study explicitly underlines that myocardial hypoperfusion upon contrast enhanced CT imaging needs to be considered as sign of acute myocardial infarction and indicates targeted clinical workup to rule out this diagnosis and to shorten the timeframe from imaging diagnosis to interventional treatment.

## Introduction

Acute myocardial infarction (AMI) is a leading cause of morbidity and mortality worldwide^1–3^. The outcome of patients with AMI is determined by multiple factors including age, comorbidities, delay to treatment and treatment strategy^4,5^. In clinical practice, timely diagnosis of AMI can be challenging in patients primarily admitted to the hospital for non-cardiac reasons and in those presenting with atypical symptoms, inconclusive ECG findings or ambiguous laboratory results^4,6^.

As per definition, the diagnosis of AMI is based on symptoms, ECG changes, cardiac troponin levels and imaging evidence of myocardial ischemia^7^. Computed tomography (CT) is an essential imaging technique which can be applied to perform non-invasive coronary angiography (CCTA). CCTA allows for the visualisation of the coronary arteries and an anatomical and functional assessment of coronary artery disease (CAD). CCTA has been proven to be a cost-effective imaging modality in patients with suspected CAD, especially in patients at low or intermediate risk^8,9^. While CCTA is mostly performed in stable patients with suspected CAD, indications for CT examinations in an emergency setting are diverse and in most cases the focus of the investigation is the exclusion of an underlying disease other than CAD or AMI^10,11^. Consequently, CT protocols are often not specifically designated for a comprehensive cardiac examination resulting in reduced visualization of cardiac structures, especially the coronary arteries^12^. On the other hand, the heart is part of the study area of both thoracic and abdominal CT imaging and as CT technology continues to evolve, knowledge of indirect signs of AMI on non-cardiac-specific emergency imaging becomes increasingly important.

In this study, we retrospectively analysed a cohort of patients with an incidental finding of suspected AMI on non-cardiac-specific emergency CT imaging, which led to further cardiological workup and invasive coronary angiography (ICA).

## Materials and methods

The ethics committee of the Christian-Albrechts-University in Kiel approved this retrospective study (AZ-D561/23). All study participants gave informed consent in a written form in accordance with the ethical standards laid down in the 1964 declaration of Helsinki and its later amendments.

### Study design and patients

This retrospective study included all consecutive patients between 2018 and 2023 who underwent ICA due to suspicion of AMI based on thoracic and/or abdominal CT imaging. In all cases, CT findings prompted further cardiological investigation leading to ICA. Patients were included in this analysis regardless of their medical history, indication for CT and the CT protocol that was used for image acquisition.

The following CT imaging findings were evaluated: (1) Presence of visible hypoattenuation in segments of the left ventricular myocardium on arterial and/or venous phase images, using the American Heart Association (AHA) 17-segment model, with their mural extent graded from 0 to 4 (0 = no hypoattenuation, 1 = hypoattenuation < 25%, 2 = hypoattenuation 25–50%, 3 = hypoattenuation 50–75%, 4 = hypoattenuation > 75%); 2) Visualisation of coronary artery occlusion, if present^13,14^. Imaging findings were evaluated by a radiologist specialized in cardiac imaging with 10 years of clinical experience (P.L.). In addition, we examined the time between end of CT image acquisition and ICA. Finally, results of ICA were correlated with the findings of the CT. Furthermore, available levels of high sensitive troponin T (hsTnT) were gathered at the time of CT imaging acquisition and after completion of ICA.

### CT imaging acquisition and image evaluation

Image acquisition was performed at our institution using three different CT scanners: Definition Flash (Siemens, Erlangen, Germany); IQon and iCT (Philips, Best, The Netherlands, respectively). The following CT settings were applied: tube voltage 100 kV, tube current time product 59 mAS, slice collimation 2.0 × 128 × 0.6 mm, pitch factor 1.2. Intravenous iodine-based contrast agent (Imeron 350, Bracco Imaging, Milano, Italy) was administered adapted to body weight (1 ml/kg; maximum 120 ml) at an injection rate of 4 ml/s. Images were analysed using multiplanar reconstruction technology as well as imaging postprocessing software (IMPAX, Agfa Healthcare, Mortsel, Belgium; SyngoVia, Siemens, Erlangen, Germany; IntelliSpace, Philips, Best, The Netherlands). ECG-triggered image acquisition was performed only in patients with suspected pathologies of the ascending aorta or CAD. The spectral CT data from the IQon CT additionally allowed material decompensation and thus the generation of iodine density images for a better visual assessment of myocardial hypoperfusion.

### Invasive coronary angiography

All interventional procedures were performed at our centre in an angiographic suite designated for percutaneous coronary interventions. Indication for and performance of ICA was at the discretion of the interventional cardiologist. Findings were retrospectively evaluated by systematic review of study reports and validated by a board-certified cardiologist with more than 10 years of clinical experience. The vascular territory involved, the presence of vascular occlusions, and the need for recanalization were noted.

### Statistical analysis

Data were processed using Microsoft Office (Microsoft, Redmond, WA, USA). Descriptive statistical analyses of the cohort were performed as indicated. Spearmann correlation was calculated to assess the agreement between hypodense myocardial segments on CT images and ICA findings. Statistical significance was set at an alpha of 0.05.

## Results

### Patients

Twenty-two patients (11 female) with suspected AMI on CT imaging were included in this study. Mean age was 66.3 ± 10.8 years. Demographics are noted in Table 1. All CT images were obtained in an emergency setting. In 16 out of 22 patients, CT was ordered to specifically exclude aortic dissection and pulmonary embolism. Furthermore, CT imaging was conducted for the assessment of inflammatory disease/sepsis, postoperative course after type-A-aortic dissection, polytrauma, cardiopulmonary resuscitation, upper abdominal pain after abdominal surgery, unclear loss of consciousness with suspicion of thoracic haemorrhage and unclear hemodynamic instability with elevated serum lactate concentrations. Exclusion of AMI was an explicit additional question for CT imaging in only two cases among exclusion of aortic dissection and pulmonary embolism.

**Table 1:** Patient demographics, Clinical and Imaging findings.

Demographics	
No. of patients	22
No. of female	11 (50%)
Mean Age	66.3 (42–79) years
Cardiovascular risk factors	
Diabetes	5 (22.7%)
Hypertension	12 (54.5%)
Hyperlipidaemia	9 (40.9%)
Smoking	5 (22.7%)
Previous MI	3 (13.6%)
Clinical and laboratory findings	
STEMI	13 (59%)
NSTEMI	9 (41%)
Mean troponin increase	5158.9 (45–24552) ng/l
Mean time between Troponin 1 and 2	420.2 (90–710) min
Norm value troponin	0–14 ng/l
CT findings	
Overall hypoperfused segements	109/374
Mean number of affected segments	5 (1–9)
Mean time to ICA	196 (36–4044) min

### CT imaging findings

In total, 109 of the 374 myocardial segments examined were classified as having impaired perfusion. On average, 5 segments per patient were visually hypoperfused compared to the surrounding healthy myocardium, ranging from 1 to 9 segments. The width of the hypoperfused segments was classified as grade 4 in 16 patients, grade 3 in four patients and grade 2 in one patient. Following the 17-segment cardiac segmentation model, the hypodense segments were located in the left anterior descending artery (LAD) territory and right coronary artery territory (RCA) in nine patients, respectively, and in the left circumflex artery (LCX) territory in four patients.

### Laboratory and ICA findings

In all patients, hsTnT levels were collected at the time of the CT image acquisition and after ICA. Mean hsTnT level at CT imaging time was 1814.1 (16.1–12502.0) ng/l and after ICA 6648.1 (61.4–27816) ng/l. A mean timeframe of 420.2 (90–710) minutes was observed between collection of hsTnT levels, whereby a mean increase of 5190 (45–24552) ng/l was seen.

All patients with suspected AMI on CT imaging were found to have lesions requiring treatment upon ICA. A total of 27 lesions were identified in the 66 vascular territories examined. In 18 patients, complete vessel occlusion was present, while the other four patients had severe stenosis. Overall, culprit lesions were identified in the LAD in eight cases and in the LCX and RCA in seven cases, respectively. Spearmann correlation with hypoperfused segments on CT imaging for the corresponding vascular territory in ICA showed a strong correlation (ρ = 0.73, p = < 0.001).

**Fig. 1:**
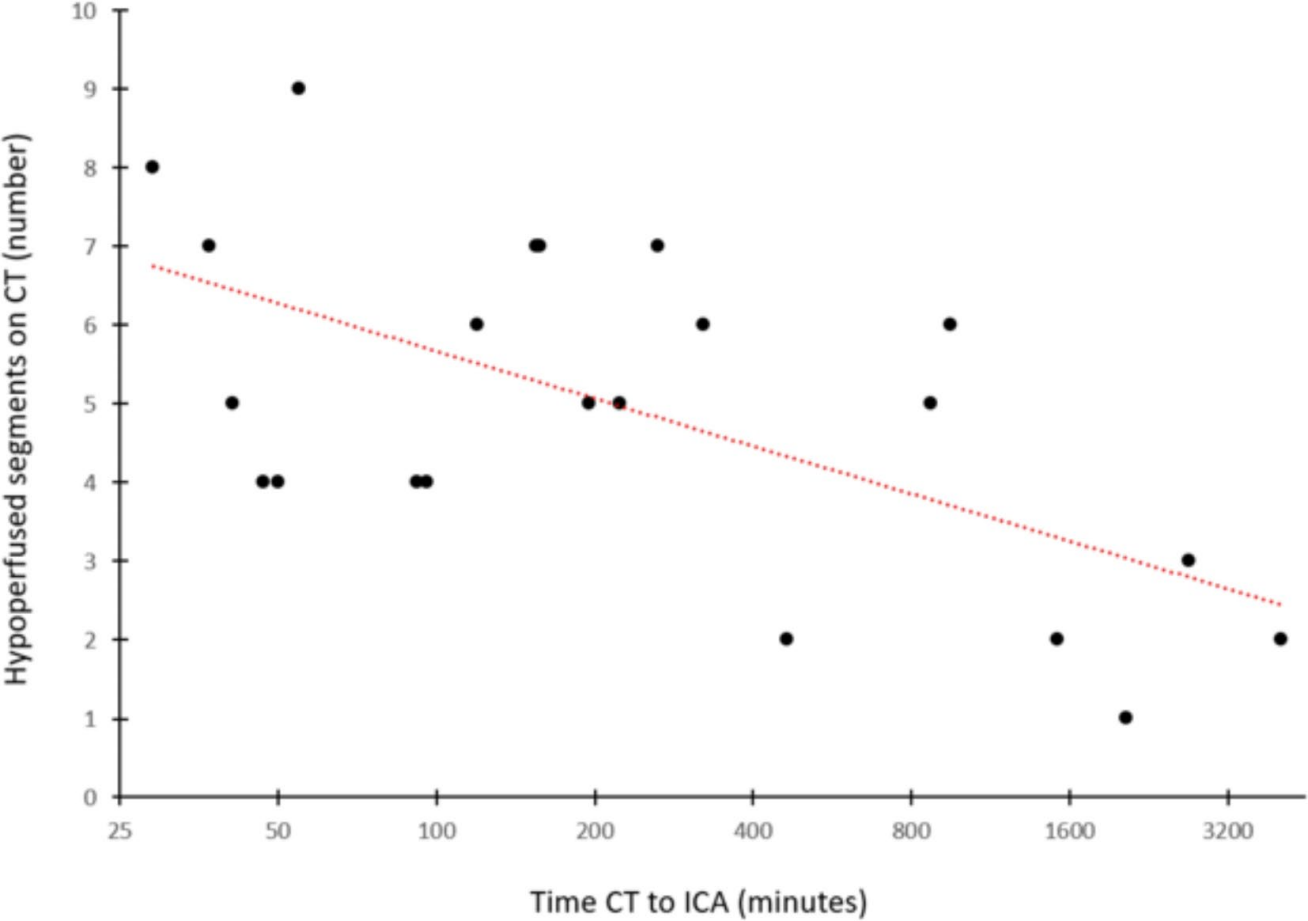
Time to ICA in dependence of number of segments with visual myocardial hypoperfusion on CT imaging. The y-axis shows the number of segments with hypoperfusion on CT imaging, while the x-axis depicts the time in minutes between the CT exam and the ICA. The red-dotted trendline within the diagram depicts that the higher the number of segments with visual hypoperfusion on imaging, the faster an ICA examination was initiated. In clinical context, this means that the more segments are seen to be affected, the more likely the patient was to develop either symptoms suggestive of AMI, distinctive ECG changes or biomarker dynamics that led to an ICA examination being performed earlier.

The mean time between the suspicion of AMI on CT imaging and ICA was 196 (37–4044) minutes. The higher the number of myocardial segments affected, the faster the ICA was begun (Fig. 1). Percutaneous coronary angiography was performed in all patients, whereby in three patients no coronary interventions were performed - twice due to distal occlusions not accessible to thrombectomy maneuvers and once due to reserved prognosis.

## Discussion

In this study, we report the results of 22 patients who underwent ICA because of suspected AMI as an incidental finding on CT. The main findings are: (1) emergency CT imaging allowed for the detection of myocardial hypoperfusion suggestive of AMI, independent of three different CT systems and without the use of cardiac-specific imaging protocols; (2) ICA confirmed significant CAD in all cases; (3) a long delay between CT and ICA was found especially in patients with a lower number of hypoperfused myocardial segments.

### CT myocardial hypoperfusion for the diagnosis of AMI

CT can provide a qualitative assessment of myocardial perfusion in a single scan phase. During the initial contrast passage in arterial phase images, patients with acute myocardial infarction will have myocardial segments with impaired perfusion which can be depicted as areas of hypoattenuation on CT imaging^15,16^. This phenomenon can also be visualised in delayed contrast phase images.

Myocardial hypoperfusion represents a visually circumscribed relative hypoattenuation of tissue affected by vascular occlusion or stenosis compared to healthy myocardium with normal perfusion.

**Fig. 2:**
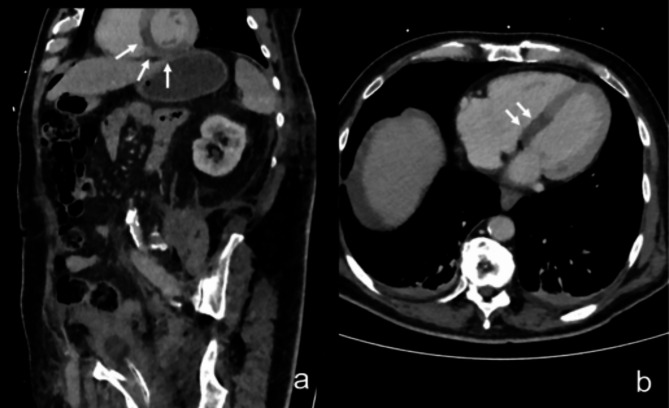
Incidental finding of myocardial hypoperfusion on venous phase abdominal CT imaging for evaluation of postoperative hemorrhage after radical prostatecomty. In this image, reconstructed abdominal CT images in oblique (**a**) and axial (**b**) view are shown. Imaging was conducted due to suspicion of abdominal bleeding after previous radical prostatectomy. Myocardial hypoperfusion located at the basal and midventricular level of the septum and the inferior wall can be delineated (white arrows, images a and b).

**Fig. 3:**
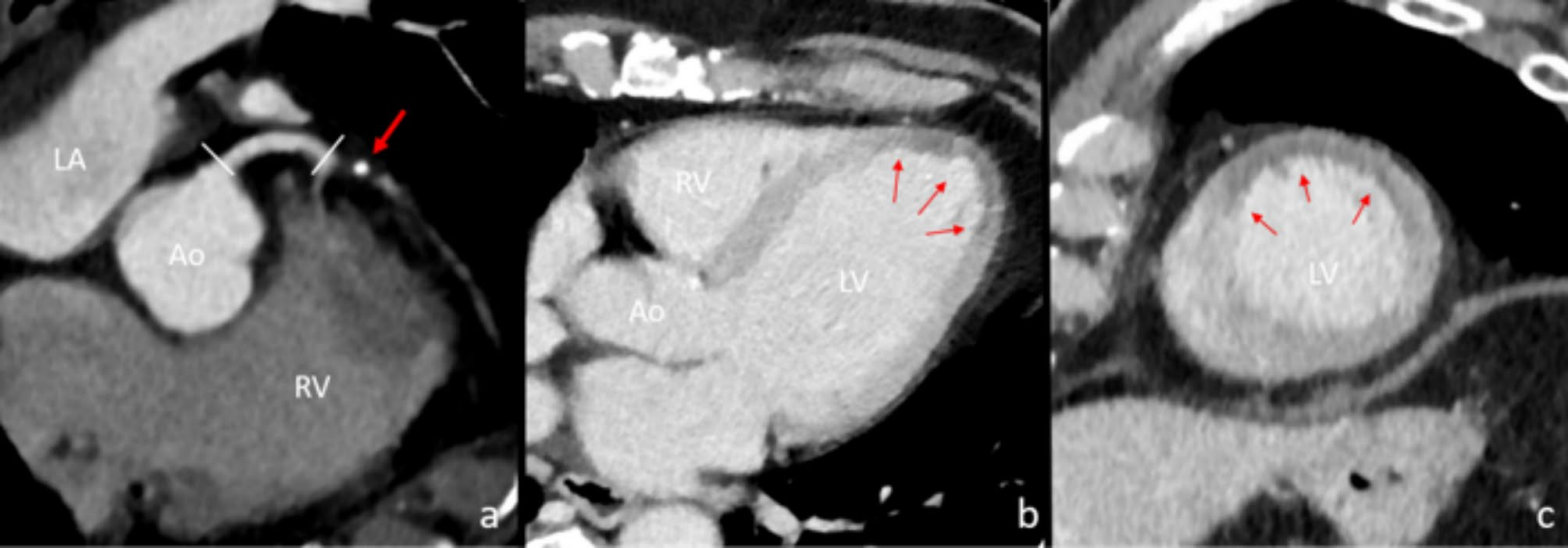
Exemplary case of proximal LAD occlusion with corresponding anterior perfusion defect. In image a, multiplanar reconstruction depicts regular contrasting of the left main coronary artery (**a**, white arrows). In the proximal LAD, a calcified plaque can be seen with only very faded contrast media in the downstream LAD. This plaque likely ruptured, subsequently causing LAD occlusion (**a**, red arrow). In images b and c, the corresponding anterior and apical perfusion defects of the left ventricular myocardium can be seen (**b** and **c**, red arrows). *Ao* Aorta, *RV* right ventricle, *LV* left ventricle.

This phenomenon occurs in contrast-enhanced CT studies and is independent of ECG-gating and contrast phase (Figs. 2 and 3). Previous studies have shown that acute myocardial infarction can be visualised on ungated contrast-enhanced chest CT as such a circumscribed hypodense myocardial area, particularly in patients undergoing imaging to rule out acute aortic dissection and pulmonary embolism^17–22^. However, these studies only retrospectively assessed CT imaging after completion of ICA studies. A recent study by Talakić et al. showed that myocardial hypoperfusion may also be visible on emergency contrast-enhanced abdominal CT and is often missed by radiologists^23^.

In our study, every patient with an initial suspicion of AMI upon imaging underwent ICA. The initial referral to CT was to rule out other thoracic and abdominal diseases as presence of AMI was not a dedicated question to imaging. Our study highlights the diagnostic ability of myocardial hypoperfusion on CT as an indicator of myocardial ischemia, which has been reported in other studies to have a sensitivity and specificity of up to 93% for AMI detection^18^.

ICA revealed either complete vessel occlusion or high-grade stenosis in all patients, leading to attempted recanalization procedures in all but three patients. In two of these patients, ≤ 2 of the 17 myocardial segments were affected on the initial CT. Delayed angiographic workup after 8 h in one case and 34 h in the other case showed only small distal occlusions that were deemed not accessible for interventional treatment. In one patient, imaging suspected AMI following successful cardiopulmonary resuscitation, but there were concurrent signs of acute upper gastrointestinal haemorrhage. In this case, acute haemorrhage with hemodynamic shock remained the working hypothesis and ICA was delayed to enable endoscopic assessment of the upper gastrointestinal tract. The subsequently conducted ICA showed non-occlusive severe CAD, but no coronary intervention was performed due to progressive septic shock and reserved prognosis.

### Clinical management after suspicion of AMI on CT imaging

In our study, the mean time between CT with an initial suspicion of AMI and the beginning of the ICA was 196 min, ranging from 34 to 4044 min. This large variability may be explained by several reasons. Firstly, specific cardiac evaluation was initiated only after myocardial hypoperfusion was diagnosed upon imaging, as AMI was not suspected before. Secondly, some patients were found to have other critical pathologies, e.g. rupture of the left ventricle and acute abdominal bleeding, that required immediate surgical treatment. Thirdly, the ICA procedures at our hospital are done in a separate building, and the required transportation to the intervention likely added to the elongated timeframe. Finally, patients who had no clinical signs of AMI besides myocardial hypoperfusion upon imaging underwent delayed ICA (Fig. 1).

### Initiation of targeted cardiologic workup and avoidance of treatment delay

In clinical practice, immediate as well as serial laboratory and ECG assessment will be conducted to exclude AMI within 1–3 h^24,25^. For patients with inconspicuous findings, further imaging with echocardiography or elective cardiac computed tomography will be initiated to rule out CAD.

**Fig. 4:**
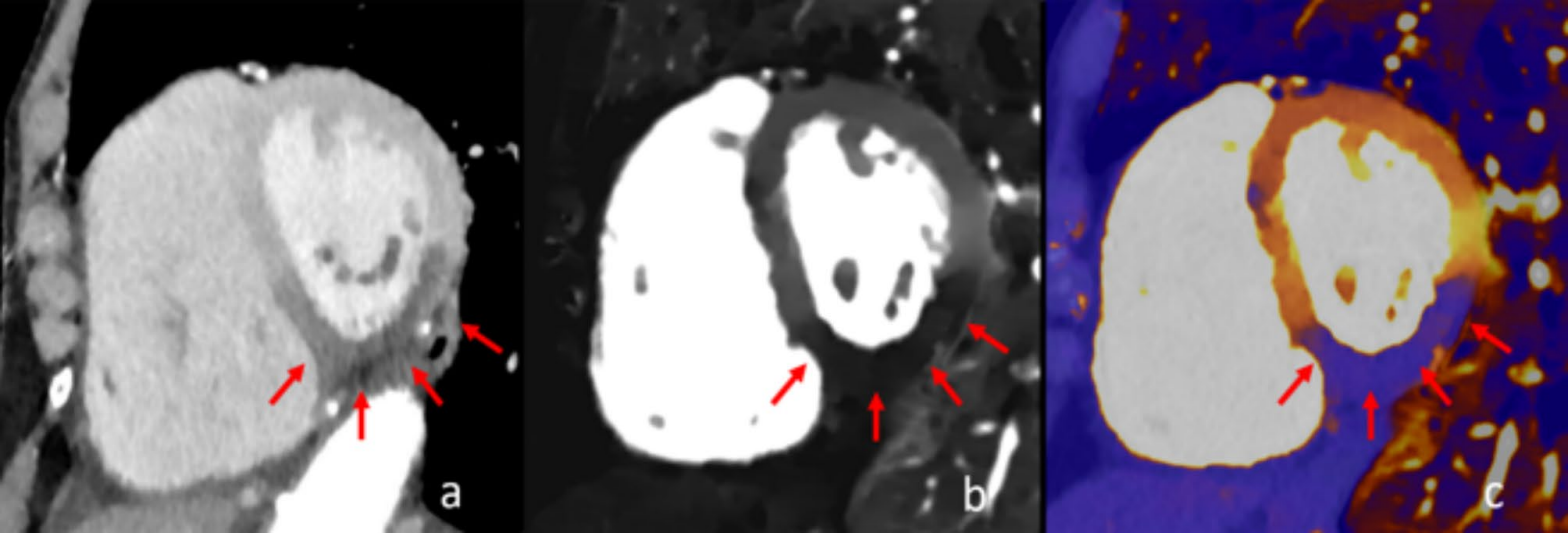
Myocardial Hypoperfusion in different reconstructions with imaging postprocessing. In images (**a**–**c**), myocardial hypoperfusion of the septal, inferior, and lateral wall at the midventricular level is depicted. While image (**a**) represents the conventional reconstruction, images (**b**) and (**c**) depict reconstructions derived from iodine density maps, where the affected segment can be delineated more clearly.

In case of AMI, revascularization therapy should be initiated as soon as possible to improve patient outcomes^4,6^. Recently, a study by Rashid et al. showed that in patients referred to ICA due to AMI, prolonged door-to-balloon time remains to be associated with an increased mortality. This emphasizes the need for improvements in CT-to-catheter time in patients with suspected AMI upon CT imaging^26^. Symptoms of AMI can be variable and even non-specific, leading to an initial suspicion of a diagnosis other than acute coronary syndrome. Regardless, CT findings suggestive of AMI should be considered as an emergency and prompt the initiation of further targeted cardiological diagnostics. Here, it will be crucial to implement strategies to optimize the CT-to-catheter time, especially in patients with large numbers of hypoperfused myocardial segments. This can be achieved by increased awareness of myocardial hypoperfusion in thoracic or abdominal CT imaging in patients presenting for evaluation of any acute pathology. Once myocardial hypoperfusion is suspected, further cardiologic workup has to be forced to possibly keep the timeframe to interventional treatment as short as possible (Fig. 4). Ongoing developments in CT technology also allow for a deeper level of assessment of cardiac diseases, including AMI, even when using non-ECG-triggered CT imaging (Figs. 3 and 4)^27^. In this context, it can be expected that the increasing use of advanced CT systems and the growing radiological education regarding the detection of myocardial ischemia on CT imaging will increase the detection rates of patients with AMI. Furthermore, detection of findings suggestive of myocardial ischemia may be an area of interest for AI-based software.

### Limitations

**Fig. 5:**
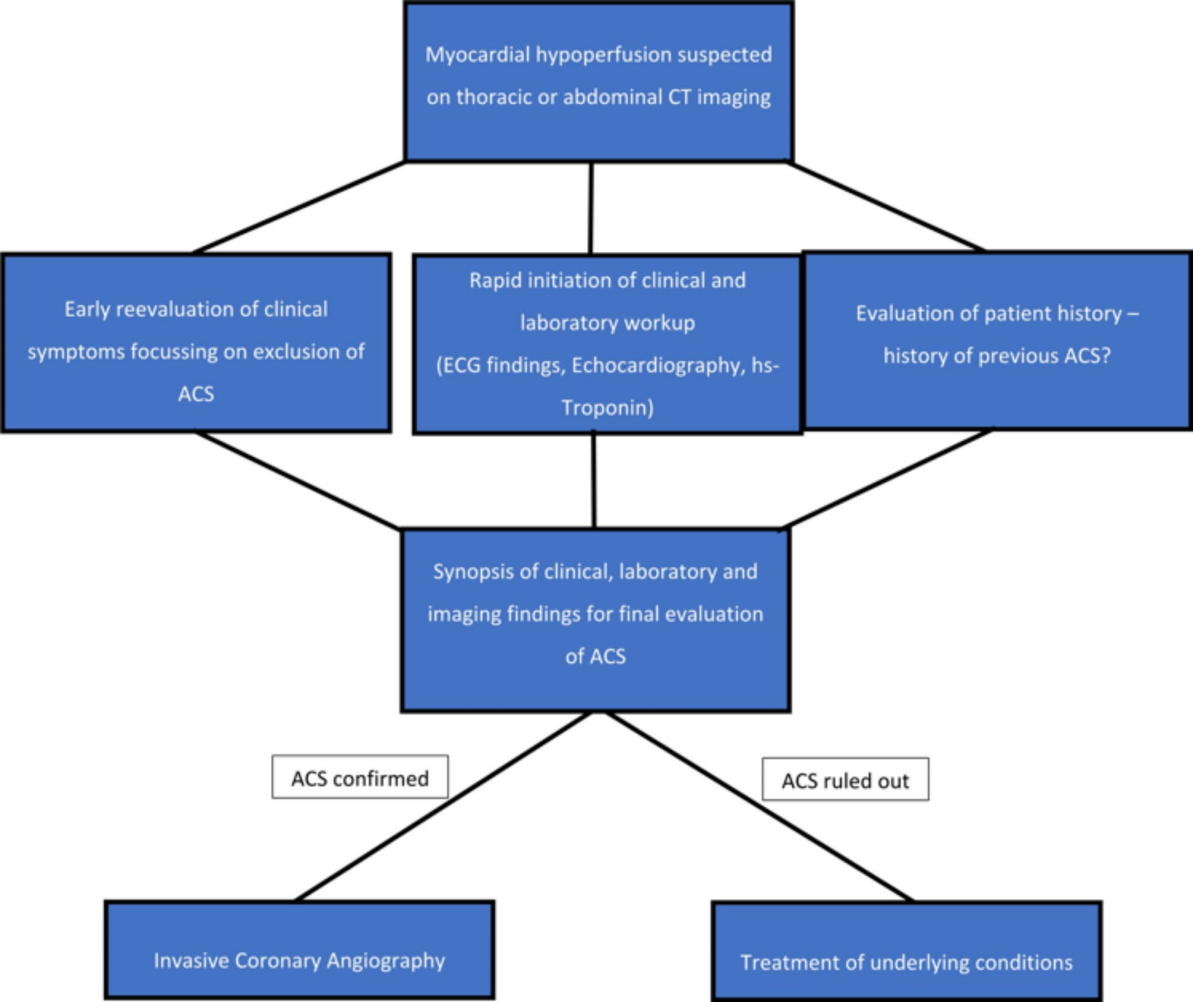
Flowchart for incidental myocardial hypoperfusion on CT imaging.

The present study has several limitations. First, this was a single-center study with a small sample size. Second, correct image interpretation concerning myocardial hypoperfusion requires experience and expertise of the radiologist in reading of cardiac imaging. False-positive CT findings were not present in our study, but myocardial hypoperfusion can occur in old myocardial infarction, which may be a source of interference. While three patients of our cohort had known previous myocardial infarction, this did not interfere in our study. Third, myocardial hypoperfusion on non-cardiac specific imaging cannot determine the acuity of myocardial infarction, which can only be interpreted in the clinical context using ECG findings and biomarker concentrations (Fig. 5). Despite these limitations our study highlights the role of CT for the detection of AMI in the emergency setting.

## Conclusion

Myocardial hypoperfusion on emergency CT imaging should be interpreted as sign indicative of acute myocardial infarction and prompt further cardiological workup, especially in patients in whom acute myocardial infarction is not primarily suspected. Improvements in diagnostic and clinical workup are needed to optimize the time period from CT imaging to invasive diagnostics and treatment. Our study points out the importance of shortening the CT—to—catheter time to enable timely treatment and to potentially improve patient outcomes.

## Data Availability

The datasets used and analysed during the current study are available from the corresponding author on reasonable request.

## Abbreviations

AMI: Acute myocardial infarction
ACS: Acute coronary syndrome
ECG: Electrocardiogram
CT: Computed tomography
cCTA: Coronary computed tomography
CAD: Coronary artery disease
mAS: Miliampere-second
kV: Kilovoltage
LAD: Left anterior descending artery
RCA: Right coronary artery
CX: Circumflex artery
